# The rs3850641 polymorphism of the TNFSF4 gene increases the risk of myocardial infarction in a Chinese Han population

**DOI:** 10.1042/BSR20180526

**Published:** 2018-07-13

**Authors:** Changqing Lu, Helei Jia, Aiguo Xu

**Affiliations:** 1 Department of Emergency, The Second Affiliated Hospital of Henan University of Chinese Medicine (Henan Province Hospital of Traditional Chinese Medicine), Zhengzhou, Henan 450002, China; 2 Department of Emergency, Henan Province Hospital of Traditional Chinese Medicine, Zhengzhou, Henan 450002, China; 3 Respiratory Intensive Care Unit, The First Affiliated Hospital Zhengzhou University, Zhengzhou 450052, China

**Keywords:** case-control study, myocardial infarction, single-nucleotide polymorphism, TNFSF4

## Abstract

Tumor necrosis factor superfamily member 4 (TNFSF4), also known as Ox40 ligand (Ox40l), plays an important role in atherosclerosis development. Several studies reported the association between the rs3850641 polymorphism of the TNFSF4 gene and the risk of myocardial infarction (MI). However, the results are inconsistent. In order to explore the relationship between the rs3850641 polymorphism of the TNFSF4 gene and MI, we conducted a case–control study including 454 cases and 512 controls in a Chinese Han population. Genotyping was performed using matrix-assisted laser desorption/ionization time-of-flight mass spectrometry. The present study found that AA genotype (AA vs. GG: odds ratio (OR) & 95% confidence interval (CI), 2.00(1.04,3.86), *P*=0.039; AA vs. AG+GG: OR & 95% CI, 1.93(1.00,3.70), *P*=0.049) or A allele carriers (A vs. G: OR & 95% CI, 1.27(1.00,1.60), *P*=0.047) of the rs3850641 polymorphism of the TNFSF4 gene increased the risk of MI. In conclusion, this case–control study confirms that the rs3850641 polymorphism of the TNFSF4 gene increases the risk of MI.

## Introduction

Coronary heart disease (CHD) is a significant risk factor for mortality, with myocardial infarction (MI) being the most serious (and fatal) consequence of CHD [1]. The main pathogenesis of MI is atherosclerosis [2], in which raised areas of degeneration and cholesterol deposits form on the inner surfaces of the arteries obstructing blood flow. Smoking, alcohol intake, diabetes, hypertension, hypercholesterolemia, obesity, physical inactivity, and certain psychosocial factors are known to be risk factors for MI pathogenesis [3]. Although chronic inflammation in MI has been studied extensively, the relationship between inflammation and MI remains unclear. However, several studies have demonstrated that genetic factors play a critical role on MI development [4,5].

Tumor necrosis factor superfamily number 4 (TNFSF4), also known as OX40 ligand (OX40L), is located in human chromosome 1 and encodes a type II glycoprotein. The expression of TNFSF4 has been observed in T cells, B lymphocytes, vascular endothelial cells, macrophages, mast cells, and smooth muscle cells, all of which are involved in the development of atherosclerosis [6,7]. Malarstig et al. [8] suggested that TNFSF4 gene polymorphisms are associated with the incident atherothrombosis and venous thromboembolism risk in Caucasians. Recently, it was also suggested that single nucleotide polymorphisms (SNPs) of the TNFSF4 gene are associated with MI and CHD severity in humans [9]. Therefore, it is reasonable to hypothesize that the TNFSF4 may be a candidate gene for MI susceptibility.

Several studies have reported an association between the rs3850641 polymorphism of the TNFSF4 gene and the risk of MI [10–14]. However, the results are contradictory. Thus, we conduct a case–control study in a Chinese Han population to investigate the relationship between rs3850641 polymorphism of the TNFSF4 gene and MI risk.

## Materials and methods

### Patients

A hospital-based case–control design was used in the present study. In total, 454 hospitalized MI patients were recruited from the First Affiliated Hospital Zhengzhou University between January 2013 and May 2017. Diagnosis of MI was confirmed by identifying stenosis in any of the major coronary arteries or in the left main trunk using coronary angiography.

In total, 512 controls were randomly selected among subjects who received regular health examinations at the First Affiliated Hospital Zhengzhou University between January 2013 and May 2017. Individuals with congestive heart failure, peripheral vascular disease, rheumatic heart disease, pulmonary heart disease, chronic kidney disease, hepatic disease, or any malignancy were excluded from the study.

Demographic, lifestyle, and clinical characteristics of all patients and controls, including age, body mass index (BMI), total cholesterol (TC), low-density lipoprotein (LDL), and high-density lipoprotein (HDL) were extracted from medical records. Written informed consent was obtained from all included patients and controls. We obtained approval for the study protocol from the Ethics Committee of the First Affiliated Hospital Zhengzhou University. The ethical approval of our study was in line with the standards of the Declaration of Helsinki.

### DNA extraction and genotyping

Blood samples were collected using vacutainer tubes and then transferred to ethylenediaminetetraacetic acid (EDTA) tubes. Genomic DNA was isolated from whole blood using the QIAamp DNA Blood Mini Kit (Qiagen, Hilden, Germany). Genotyping was performed by matrix-assisted laser desorption/ionization time-of-flight mass spectrometry (MALDI-TOF MS) as previously described [15]. SNP genotyping was performed using the MassARRAY system (Sequenom, San Diego, California) and the MALDI-TOF MS method. To ensure the quality of genotyping, the MALDI-TOF MS method was performed without knowledge of patient status (case vs. control).

### Statistical analysis

Demographic characteristics and rs3850641 polymorphism genotypes of the TNFSF4 gene were evaluated using a chi-squared (χ^2^) test (for categorical variables) and Student’s *t*-test (for continuous variables). The associations between the rs3850641 polymorphism A/G genotypes of the TNFSF4 gene and the risk of RA were estimated by calculating odds ratios (ORs) and 95% confidence intervals (CIs) using logistic regression analysis, and by crude ORs and adjusted ORs when adjusting for age and sex. The Hardy–Weinberg equilibrium (HWE) was assessed by a goodness-of-fit χ^2^ test to compare the observed genotype frequencies with the expected frequencies in controls. The present study was powered to detect the effect of the rs3850641 polymorphism of the TNFSF4 gene on MI susceptibility at a *P* value of 0.05 [16]. All statistical analyses were performed using the SAS software package (ver. 9.1.3; SAS Institute, Cary, NC, U.S.A.).

## Results

### Characteristics of the study population

The characteristics of the subjects in the case and control groups are summarized in Table 1. The average age was 61.04 years and 63.2% of the MI patients were men. In the control group, the average age was 61.05 years and 64.8% of the patients were men. The groups were well matched, with no significant differences in gender and age being observed between the patients and controls. TC, HDL, and LDL values are listed in the left column. The average blood pressure, BMI, TC, and LDL were significantly higher in MI patients compared with controls, while HDL was lower. These results demonstrated that hypertension, hyperlipidemia, and obesity are important risk factors for the development of MI in the Chinese Han population.

**Table 1:** Patient demographics and risk factors in myocardial infarction

Variable	Cases (*n*=454)	Controls (*n*=512)	*P*
Age (years)	61.04 ± 10.81	61.05 ± 11.01	0.990
Female/male	167/287	180/332	0.599
Smoking (No/Yes)	184/270	358/154	<0.001
Drinking (No/Yes)	329/125	436/76	<0.001
Diabetes (No/Yes)	295/159	435/77	<0.001
FPG (mmol/l)	6.40 ± 1.65	5.65 ± 1.46	<0.001
Hypertension (No/Yes)	185/269	300/212	<0.001
BMI (kg/m^2^)	27.77 ± 3.11	25.50 ± 3.13	<0.001
Total cholesterol (mmol/l)	4.81 ± 1.05	4.52 ± 1.05	<0.001
HDL (mmol/l)	1.13 ± 0.33	1.61 ± 0.39	<0.001
LDL (mmol/l)	2.89 ± 0.88	2.69 ± 0.75	<0.001

Abbreviations: BMI, body mass index; FPG, fasting blood glucose; HDL, high-density lipoprotein; LDL, low-density lipoprotein.

### Association between TNFSF4 gene rs3850641 polymorphism and MI risk

The genotype distributions of the rs3850641 polymorphism of the TNFSF4 gene among all subjects are shown in Table 2. Genotype distributions for the rs3850641 polymorphism in the controls conformed to HWE. Logistic regression analyses revealed that the rs3850641 polymorphism increased the risk of MI in three genetic models (GG vs. AA: adjusted OR = 2.29, 95% CI: 1.20–4.69, *P*=0.023; GG vs. AG+AA: adjusted OR = 2.25, 95% CI: 1.10–4.57, *P*=0.026; G vs. A: OR = 1.27, 95% CI: 1.00–1.46, *P*=0.047) (Table 2). However, we found no significant association between genotype and the clinical or biochemical characteristics (Table 3). We further evaluated the effects of the SNP on MI risk according to patient characteristics, including age, gender, and smoking, drinking, and diabetes status. The increased MI risk conferred by rs3850641 was more significant in male, nonsmoking, and nondrinking patients (Table 4).

**Table 2:** Logistic regression analysis of associations between TNFSF4 gene rs3850641 polymorphism and the risk of myocardial infarction

Genotype	Cases^1^ (*n*=454)		Controls^1^ (*n*=512)		OR (95% CI); *P*	Adjusted OR^2^ (95% CI); *P*
	*n*	%	*n*	%		
AG vs. AA	128/300	28.2/66.1	135/360	26.3/70.3	1.14 (0.86,1.52); 0.377	1.07 (0.78,1.47);0.665
GG vs. AA	25/300	5.5/66.1	15/360	2.9/70.3	**2.00 (1.04,3.86);** 0.039	**2.29 (1.20,4.69);0.023**
GG+AG vs. AA	153/300	33.7/66.1	150/360	29.2/70.3	1.22 (0.93,1.61); 0.146	1.19 (0.88,1.60);0.264
GG vs. AG+AA	25/428	5.5/94.3	15/495	2.9/96.6	**1.93 (1.00,3.70);** 0.049	**2.25 (1.10,4.57);0.026**
G vs. A	178/728	19.6/80.2	165/855	16.1/83.5	**1.27 (1.00,1.60);** 0.047	

Bold values are statistically significant (*P*<0.05).

1 The genotyping was successful in 453 cases and 510 controls.

2 Adjusted for age, sex, smoking, drinking, hypertension, diabetes.

**Table 3:** The clinical and biochemical characteristics of *TNFSF4* rs3850641 polymorphism among two groups

	Patients (*N*=454)				Controls (*N*=512)			
	AA (*N*=300)	AG (*N*=128)	GG (*N*=25)	*P*	AA (*N*=360)	AG (*N*=135)	GG (*N*=15)	*P*
Age (years)	60.92 ± 10.51	61.84 ± 11.51	58.72 ± 10.75	0.392	61.08 ± 11.03	60.47 ± 11.08	63.87 ± 9.96	0.512
BMI (kg/m^2^)	27.65 ± 3.08	27.99 ± 3.01	28.06 ± 3.83	0.514	25.63 ± 3.17	25.07 ± 3.08	26.11 ± 2.67	0.167
TC (mmol/l)	4.84 ± 1.06	4.77 ± 1.06	4.67 ± 0.89	0.648	4.52 ± 1.05	4.48 ± 1.03	4.76 ± 1.09	0.622
HDL (mmol/l)	1.13 ± 0.31	1.12 ± 0.34	1.13 ± 0.43	0.987	1.62 ± 0.39	1.61 ± 0.39	1.56 ± 0.48	0.839
LDL (mmol/l)	2.94 ± 0.89	2.81 ± 0.85	2.78 ± 1.00	0.309	2.69 ± 0.75	2.71 ± 0.77	2.70 ± 0.58	0.967

Abbreviations: BMI, body mass index; HDL, high-density lipoprotein; LDL, low-density lipoprotein; TC, total cholesterol.

**Table 4:** Stratified analyses between TNFSF4 rs3850641 polymorphism and the risk of myocardial infarction

Variable	TNFSF4 rs3850641 (case/control)			GG vs. AA	GG+AG vs. AA	GG vs. AG+AA
	GG	AG	AA			
Sex						
Male	17/8	83/89	186/235	**2.68 (1.13,6.36);0.025**	1.30 (0.93,1.83);0.127	**2.56** (**1.09,6.02);0.031**
Female	8/7	45/46	114/125	1.25 (0.44,3.57);0.672	1.10 (0.69,1.73);0.693	1.23 (0.44,3.47);0.697
Age						
<60	12/6	51/64	139/164	2.36 (0.86,6.45);0.094	1.06 (0.71,1.60);0.773	2.40 (0.88,6.52);0.086
≥60	13/9	77/71	161/196	1.76 (0.73,4.22);0.206	1.37 (0.95,1.98);0.092	1.62 (0.68,3.86);0.276
Smoking status						
Nonsmoker	14/14	47/92	122/251	2.06 (0.95,4.45);0.067	1.18 (0.81,1.74);0.386	2.03 (0.95,4.35);0.069
Smoke	11/1	81/43	178/109	6.74 (0.86,52.90);0.070	1.28 (0.83,1.97);0.621	6.46 (0.83,50.49);0.076
Drinking status						
No	15/14	99/105	214/315	1.39 (1.00,1.92);0.233	**1.41 (1.03,1.92);0.030**	1.44 (0.68,3.02);0.338
Yes	10/1	29/30	86/45	5.23 (0.65,42.18);0.120	0.66 (0.36,1.19);0.168	6.52 (0.82,52.00);0.070
Hypertension						
No	10/7	50/85	125/207	0.97 (0.64,1.47);0.974	1.08 (0.73,1.60);0.702	2.38 (0.89,6.38);0.084
Yes	15/8	78/50	175/173	2.37 (0.88,6.37);0.089	1.40 (0.95,2.08);0.092	1.50 (0.63,3.62);0.362
Diabetes						
No	18/11	81/114	195/308	**2.59 (1.20,5.59);0.016**	1.25(0.91,1.72);0.169	**2.50 (1.16,5.38);0.019**
Yes	7/4	47/21	105/52	0.87 (0.24,3.09);0.826	1.07 (0.60,1.91);0.820	0.84 (0.24,2.96);0.797

Bold values are statistically significant (*P*<0.05).

### Power analysis

To investigate whether the power of the present study was sufficient given the sample size, we calculated the power of the rs3850641 polymorphism in the allelic model. Analysis showed that our study had a power of 72.1% to detect the effects of the rs3850641 polymorphism on MI susceptibility, assuming an OR of 1.27.

## Discussion

In the present study, we investigated the association between the rs3850641 polymorphism of the TNFSF4 gene and the risk of MI in a Chinese population and found that the polymorphism conferred an increased risk of MI.

TNFSF4 is a T-cell activating factor that seems to facilitate the survival and/or promote anti-CD3-induced CD4+ T cells proliferation at the time of inflammation [7]. T cells may play an important role in the development of atherosclerosis [17]. TNFSF4 is expressed in activated vascular endothelial cells, CD4+ and CD8+ T cells, and B cells [6]. The result of the present study agrees with one previous study conducted by Wang et al. [10], which showed that the minor allele of rs3850641 was significantly more frequent in individuals with MI than in the controls in two independent populations from Sweden. However, two studies from German and Switzerland showed no evidence of an association between rs3861950 polymorphism and MI risk [11,12]. Two Chinese studies also showed no significant difference between cases and controls in two Chinese Han populations [13,14]. Many reasons could underlie these discrepancies. First, genetic heterogeneity may exist among populations and living environments, and MI develops as a result of intricate interactions between a variety of susceptibility genes and environmental factors [18]. The effects of some genetic variants may vary across different populations and environments. Second, there were differences in design among previous studies. The Swedish case–control [10] studies were based on a systematic recruitment of individuals with incident MI admitted to coronary care units. Age- and gender-matched control subjects were selected at random among the general population. In contrast, the cases and controls enrolled in the German study [11] were recruited among subjects examined by coronary angiography at two referral centers, with controls being defined as individuals with no history of MI. Controls were not matched for age and sex. The German study included a higher proportion of females in the control group compared with the MI group, and the controls were also younger. Cases in the Swedish study were also considerably younger than cases in the German study (mean age of the two Swedish case groups was 52 and 59 years, compared with 64 years for the German cases). Third, the discrepancy may be explained by clinical heterogeneity among the studies. Fourth, the sample sizes of some studies were insufficiently large to draw a convincing conclusion; therefore, the results of some of the studies may have been false-positive or -negative due to limited sample sizes.

In the stratification analyses, we found that the risk of MI conferred by the rs3850641 polymorphism of the TNFSF4 gene remained significant in the male, nonsmoking, and nondrinking subgroups. This can be explained by the concept that susceptible individuals are likely to have a degree of exposure to risk factors. However, given the decreased sample sizes in the stratification analyses and the limited power, the results should be interpreted with caution. Despite this, our findings still provide evidence for a possible interaction between the SNP and certain MI risk factors.

Several potential limitations of this case–control study should be considered. First, the patients and controls were recruited from hospitals and may not be representative of the general population. Nonetheless, the genotype distribution of the controls was in HWE. Second, a single case–control study may not be sufficient to fully interpret the relationship between the rs3850641 polymorphism of the TNFSF4 gene and susceptibility to MI, because of the limited sample size. Larger numbers of subjects are necessary to confirm our findings. Third, we did not obtain detailed information about MI severity and response to treatment, which restricted our analyses. Fourth, the risk of MI cannot be attributed to a single TNFSF4 gene SNP; other SNPs in the TNFSF4 and other genes, as well as certain environmental factors, should also be considered. Fifth, the underlying mechanisms of this SNP in MI should also be investigated. Finally, further studies in different population may establish the true significance of the association between this SNP and MI risk.

In conclusion, our study provides strong evidence that the rs3850641 polymorphism of the TNFSF4 gene may contribute to MI risk. However, our results were obtained in a sample of limited size, and this therefore represents a preliminary conclusion. Validation through multicenter case–control studies with diverse ethnic populations is needed to confirm our finding.

## Abbreviations

BMI: body mass index
CHD: coronary heart disease
CI: confidence interval
HDL: high-density lipoprotein
HWE: Hardy–Weinberg equilibrium
LDL: low-density lipoprotein
MI: myocardial infarction
OR: odds ratios
SNP: single nucleotide polymorphism
TC: total cholesterol
TNFSF4: tumor necrosis factor superfamily member 4
